# Colonization with resistant microorganisms in patients transferred from abroad: who needs to be screened?

**DOI:** 10.1186/s13756-015-0071-6

**Published:** 2015-07-25

**Authors:** T. Kaspar, A. Schweiger, S. Droz, J. Marschall

**Affiliations:** Department of Infectious Diseases and Prevention, Inselspital, Bern University Hospital, University of Bern, Bern, Switzerland; Department of Internal Medicine, University Hospital Basel, Basel, Switzerland; Institute for Infectious Diseases, University of Bern, Bern, Switzerland

**Keywords:** Screening, Multi-drug resistant organisms, Gram–negative bacteria, Colonization, International travel

## Abstract

**Background:**

While multi-drug resistant organisms (MDRO) are a global phenomenon, there are significant regional differences in terms of prevalence. Traveling to countries with a high MDRO prevalence increases the risk of acquiring such an organism. In this study we determined risk factors for MDRO colonization among patients who returned from a healthcare system in a high-prevalence area (so-called transfer patients). Factors predicting colonization could serve as screening criteria to better target those at highest risk.

**Methods:**

This screening study included adult patients who had been exposed to a healthcare system abroad or in a high-prevalence region in Switzerland over the past six months and presented to our 950-bed tertiary care hospital between January 1, 2012 and December 31, 2013, a 24-month period. Laboratory screening tests focused on Gram-negative MDROs and methicillin-resistant *Staphylococcus aureus* (MRSA).

**Results:**

A total of 235 transfer patients were screened and analyzed, of which 43 (18 %) were positive for an MDRO. Most of them yielded Gram-negative bacteria (42; 98 %), with only a single screening revealing MRSA (2 %); three screenings showed a combination of Gram-negative bacteria and MRSA. For the risk factor analysis we focused on the 42 Gram-negative MDROs. Most of them were ESBL-producing *Escherichia coli* and *Klebsiella pneumoniae* while only two were carbapenemase producers. In univariate analysis, factors associated with screening positivity were hospitalization outside of Europe (*p* < 0.001), surgical procedure in a hospital abroad (*p* = 0.007), and - on admission to our hospital - active infection (*p* = 0.002), antibiotic treatment (*p* = 0.014) and presence of skin lesions (*p* = 0.001). Only hospitalization outside of Europe (Odds Ratio, OR 3.2 (95 % CI 1.5- 6.8)) and active infection on admission (OR 2.7 (95 % CI 1.07- 6.6)) remained as independent predictors of Gram-negative MDRO colonization.

**Conclusion:**

Our data suggest that a large proportion of patients (i.e., 82 %) transferred to Switzerland from hospitals in high MDRO prevalence areas are unnecessarily screened for MDRO colonization. Basing our screening strategy on certain criteria (such as presence of skin lesions, active infection, antibiotic treatment, history of a surgical procedure abroad and hospitalization outside of Europe) promises to be a better targeted and more cost-effective strategy.

## Introduction

Multi-drug resistant organisms (MDRO) are known to negatively impact patient outcomes (due to delaying and limiting antibiotic treatment options) and represent a considerable threat to public health because they can be transmitted from person-to-person. MDROs are becoming more prominent worldwide [1, 2]. In Europe, the average percentage of *Klebsiella pneumoniae* resistant to third generation cephalosporins (a surrogate for the production of extended-spectrum betalactamase, ESBL) increased markedly over the last few years (21.5 % in 2009 to 25.7 % in 2012) [3]. Nevertheless, there are significant regional differences in prevalence [1–4]. In 2012, the percentage of *K. pneumoniae* resistant to third generation cephalosporin for example was 1.7 % in Finland, 10-25 % in Germany and France, 25-50 % in Italy and 74.8 % in Bulgaria [3]. Certain countries outside Europe suffer from enormous rates of MDROs (e.g., India, with an average prevalence of 72 %) [1]. In Switzerland, the “Sentinel Surveillance of Antibiotic Resistance in Switzerland” (www.anresis.ch) identified 5.4 % of *K.pneumoniae* to be resistant to third generation cephalosporins in 2012 [5]. In our own institution, only 0.9 % of the *K. pneumoniae* tested in 2012 were ESBL producers.

MDROs may simply colonize a patient or cause an infection. Delayed administration of adequate antibiotic therapy and increased mortality are consequences of infection with MDROs [6, 7]. Being colonized with an MDRO alone may lead to prolonged ICU stays, however, and is associated with increased mortality [8]. Apart from the implications for individual patients, there is the potential for interpersonal transmission and outbreaks [2, 9, 10]. These transmissions do not stop at international borders. Patients often return either colonized or infected with an MDRO after hospitalization abroad [2, 8, 11–13] especially if the visited country struggles with antimicrobial resistance. A good example for this is the spread of NDM-1 (New Delhi metallo-β-lactamase): this resistance gene was first detected in 2008 in a patient who had been hospitalized in India; today, just a few years later, NDM-1 has spread to forty countries [13]. The stay in regions with a high MDRO prevalence is therefore an established risk factor for the acquisition of such an organism [12, 14, 15].

The Infection Prevention Unit at Bern University Hospital pursues a multi-pronged strategy to keep the MDRO prevalence low that is based on recommendations by national and international guidelines [16–18]. One goal of this strategy is to identify patients who are colonized with a MDRO as early as possible. This aligns with recommendations to screen those with a high risk of being colonized with an MDRO [15, 19], including patients transferred directly from a hospital abroad or those who were hospitalized abroad in the previous four weeks up to 12 months [15, 20]. Due to limited data on the MDRO situation in many countries a German expert commission now advises to screen patients with a suggestive medical history irrespective of the place of stay abroad [15].

The aim of our study was to determine risk factors for MDRO colonization among transfer patients that will help us target high-risk patients in our future approach to screening.

## Methods

The University Hospital of Bern is a 950-bed tertiary care hospital that includes a 30-bed intensive care unit (ICU) and covers all medical specialties. Each year, there are approximately 38`000 admissions. According to written local infection control guidelines so-called “transfer patients” are screened on admission for carriage of MRSA (methicillin-resistant *Staphylococcus aureus*), ESBL (extended spectrum betalactamase) -positive, and carbapenemase-producing Gram-negative bacteria. Transfer patients are defined as patients who were exposed to a healthcare system abroad or in a high prevalence region in Switzerland over the past six months. High prevalence regions in our country are those with ESBL *K. pneumoniae* or MRSA rates >10 %, such as western and southern Switzerland (www.anresis.ch; accessed March 20, 2015). Exposure to a healthcare system includes admission to a hospital or outpatient visit in a medical center independent of the time the patient spent there or the reason for seeking care. The definition includes both patients who were directly transferred from a hospital abroad (i.e., repatriated) and those who returned by themselves. Transfer patients are screened at our institution regardless of whether they were hospitalized or seen as outpatients.

In this screening study we analyzed transfer patients who had a *standard admission screening* (with or without additional screening tests) between January 1, 2012 and December 31, 2013 (a 24-month period). A standard admission screening for MDRO required at minimum a nasal and an inguinal swab plus either a rectal swab or a stool sample. Additionally, if present, a swab was taken from skin lesions, wound drainage in a patient with a drain in place, urine in patients with urinary catheter and tracheal secretions in intubated or tracheostomy patients; these risk factor-based screening tests were labeled as *extended screening*. For the swabs, we used a cotton tip moistened with sterile 0.9 % saline solution. A positive admission screening meant that at least one of the screening samples showed an MDRO. A standard screening set costs approximately 118 Swiss Francs.

For identifying patients with an MDRO screening on admission, we used the database of the Bern University Institute for Infectious Diseases, where all microbiological samples from the University Hospital are processed. This dataset was supplemented with data on patients’ length of stay in the hospital abroad, the interval between that stay and our screening, country of exposure, mode of patient transfer, antibiotic treatment, hospital service at admission, medical devices at time of admission, skin lesions at admission, clinical presentation and history of surgical procedure from the hospital electronic medical records. If a patient was admitted more than once during the study period, only the first admission was included. We excluded screenees younger than 16 years.

### Microbiological analysis

For the MRSA screening we used a Mannitol-oxacillin biplate. Identification of *Staphylococcus aureus* was done according to laboratory standard procedures. Susceptibility testing of *S. aureus* was performed using the Kirby-Bauer disk diffusion test and test results were interpreted according to the Clinical Laboratory Standards Institute (CLSI) standards [21, 22].

For the screening of ESBL and carbapenemase producing bacteria we used CHROMagar™ ESBL, McConkey with ceftazidime, and Drigalski with cefotaxime [23].

### Statistical analyses

The statistical analyzes were performed using the STATA 12 software package. Categorical data were analyzed using the χ ^2^-test or the Fisher's exact test. For comparing of continuous variables we employed Student's *t*-test or Mann-Whitney *U*-test as appropriate. We created a multivariate model using logistic regression to identify independent risk factors for MDRO carriage. For assessing model quality, we used goodness-of-fit tests. A p-value of <0.05 was considered statistically significant.

### Ethical aspects

The data for patients included here were generated during routine medical care. MDRO screening was started as part of the hospital`s risk assessment strategy by the infection prevention program. There were no study-specific interventions administered. Quality improvement activities such as those initiated by the infection prevention program are exempt from IRB review at our institution.

## Results

Among 287 patients who had a complete admission screening for MDRO between January 1, 2012 and December 31, 2013, 52 did not qualify as transfer patients by our criteria and were therefore excluded. We further analyzed the remaining 235 patients. Of those, 97 (41 %) were female and 138 (59 %) men. Median age was 58 years (range, 17-95). Two hundred and fifteen (91 %) patients had their residence in Switzerland at the time of screening while 20 (9 %) patients reported a residence abroad. Of the 235 patients, 15 (6.4 %) were transferred from a hospital in a high endemic region in Switzerland, 145 (61 %) patients had contact to a healthcare system in another European country. The remaining 75 patients (32 %) returned from countries outside of Europe.

In total, 43 (18 %) of the 235 patients were screened positive for an MDRO. Most of them yielded resistant Gram-negative bacteria (42; 98 %) with only a single screening result revealing MRSA (2 %); three screenings showed a combination of MRSA and Gram-negatives. Thirty-six patients (15 %) were colonized with one bacterial species, 6 (2.6 %) with two species, and one patient (0.4 %) with three different species.

Next we attempted to determine risk factors for having a positive screening result (Table 1). For this particular analysis we focused on Gram-negative bacteria, which were detected in 42 out of 235 patients. Most of them were ESBL-positive *E. coli* and *K. pneumoniae* while only two were carbapenemase producers. Significant associations for screening positivity were found for the followings variables: antibiotic treatment on admission was more frequent in carriers vs. non-carriers [17/42 (40.5 %) carriers vs. 43/193 (22.3 %) non-carriers; *p* = 0.014], patients who had an active infection on admission [15/40 (37.5 %) vs. 29/183 (15.83 %); *p* = 0.002]. Also, skin lesions on admission were associated with a positive screening [17/42 (40.5 %) vs. 34/193 (17.6 %); *p* = 0.001]. Carriers were more likely to have undergone a surgical procedure in a hospital abroad than non-carriers [15/42 (35.7 %) vs. 33/193 (17.1 %); *p* = 0.007].

**Table 1:** Risk factors for colonization with Gram-negative bacteria in 235 transfer patients

Variable*	Positive N	Negative N	Univariate analysis odds Ratio (95%CI)	*p* value	Multivariate analysis odds Ratio (95%CI)	*p* value
sex (female)	20 (47.6 %)	77 (39.9 %)	1.4 (0.7-2.7)	0.36		
Age	53 (±16.6)	55.4 (±19.6)	0.99 (0.98-1.01)	0.46		
Swiss residents	37 (88.1 %)	178 (92.2 %)	0.6 (0.2-1.8)	0.37		
Inpatient hospitalization in Bern	36 (85.7 %)	170 (88.1 %)	0.8 (0.3-2.1)	0.67		
• Intensive care unit	10 (23.8 %)	29 (15.0 %)	1.8 (0.8-3.98)	0.17		
• surgical wards	13 (31.0 %)	74 (38.3 %)	0.7 (0.4-1.5)	0.37		
• medical wards	13 (31.0 %)	67 (34.7 %)	0.8 (0.4-1.7)	0.64		
Number of swabs/samples	4 (3-10)	3 (3-9)	1.4 (1.1-1.7)	0.03		
Risk factors on admission:						
Medical device	13 (31.0 %)	41 (21.2 %)	1.7 (0.8-3.5)	0.18		
• urinary catheter	10 (23.8 %)	36 (18.7 %)	1.4 (6.1-3.02)	0.45		
• intubation/ tracheostomy	6 (14.3 %)	11 (5.7 %)	2.8 (0.96-7.9)	0.09		
• wound drain	3 (7.1 %)	4 (2.1 %)	3.6 (0.8-16.9)	0.11		
Skin lesion	17 (40.5 %)	34 (17.6 %)	3.2 (1.5-6.5)	0.001	1.9 (0.7-4.7)	0.18
Number of risk factors	0 (0-3)	0 (0-3)	2.02 (1.4-2.97)	0.0006		
Antibiotic treatment	17 (40.5 %)	43 (22.3 %)	2.4 (1.2-4.8)	0.014	1.3 (0.5-3.3)	0.54
• number of antibiotics	0 (0-3)	0 (0-3)	1.7 (1.2-2.6)	0.008		
• antibiotic days up to admission	8.5 (±11.7)	5.8 (±6.4)	1.03 (0.96-1.1)	0.31		
Active infection	15 (37.5 %)	29 (15.9 %)	3.2 (1.5-6.8)	0.002	2.7 (1.07-6.6)	0.035
Surgical procedure abroad	15 35.7 %	33 17.1 %	2.7 (1.3-5.6)	0.007	1.5 (0.6-3.8)	0.39
Direct transfer	24 (57.1 %)	123 (63.7 %)	0.8 (0.4-1.5)	0.42		
Indirect transfer, days between last exposure abroad until admission	22 (±24.6)	21 (±28.9)	1.0 (0.98-1.02)	0.92		
Individual transportation	13 (31.0 %)	55 (28.5 %)	1.1 (0.5-2.3)	0.75		
Europe	18 (42.9 %)	142 (73.6 %)	0.3 (0.1-0.5)	<0.001		
• Switzerland	2 (4.8 %)	13 (6.7 %)	0.7 (0.2-3.2)	1		
• Spain	4 (9.5 %)	25 (13.0 %)	0.7 (0.2-2.2)	0.54		
• Italy	2 (4.8 %)	27 (14.0 %)	0.3 (0.1-1.4)	0.099		
• France	4 (9.5 %)	15 (7.8 %)	1.2 (0.4-3.97)	0.754		
Outside Europe	24 (57.1 %)	51 (26.4 %)	3.7 (1.9-7.4)	<0.001	3.2 (1.5-6.8)	0.002
• Asia	14 (33.3 %)	25 (13.0 %)	3.4 (1.6-7.2)	0.001		
o south/southeast Asia	9 (21.4 %)	11 (5.7 %)	4.5 (1.7-11.7)	0.003		
• Africa	6 (14.3 %)	11 (5.7 %)	2.8 (0.96-7.9)	0.09		
• America (North, South)	3 (7.1 %)	13 (6.7 %)	1.1 (0.3-3.9)	1		

*Mean (± standard deviation, SD) for normal distribution or median (range) for others

Note. Transfer patients are patients who were exposed to a healthcare system abroad or in a high prevalence region in Switzerland over the past six months

A standard screening set required a minimum of three samples. Those who were found to be colonized had a median of four samples (range, 3-10) which was more than the 3 samples (range, 3-9) in those with negative screenings (*p* = 0.03).

The 235 patients had contact to healthcare systems in a total of 62 different countries. Because of the low number of patient transfers from each individual country, we grouped them based on regions (Table 1). There were more carriers among those patients who had contact with a healthcare system outside of Europe than in those who returned from an European country [24/42 (57.1 %) vs. 51/193 (2.4 %); *p* < 0.001].

Of note, direct transfer form a referring hospital was not associated with a higher likelihood of colonization (*p* = 0.42). The maximum interval between last healthcare exposure abroad and presentation to our hospital was 126 days in those who returned by themselves (indirect transfer patients). Among indirect transfer patients, this interval was not different between carriers and non-carriers [21.92 (±24.6) vs. 21.16 (±28.9); *p* = 0.9]. There was also no association between screening positivity and whether patients had been in- or outpatients in a hospital abroad (*p* = 0.5).

Further, we determined independent predictors for screening positivity and identified active infection on admission as a significant risk factor [odds Ratio, OR 2.67 (95 % CI 1.07- 6.65); *p* = 0.035] as well as contact with a healthcare system outside of Europe [OR 3.23 (95 % CI 1.54- 6.79); *p* = 0.002] (Table 1).

## Discussion

This study focused on MDRO colonization in transfer patients from abroad admitted to our institution. Our data show that a significant proportion (18 %) of patients who were exposed to a healthcare system abroad or had been hospitalized in high endemicity region within Switzerland over the last six month were colonized with an MDRO. This observation reflects other reports [8, 11, 20], including two Swiss studies that found similar rates of MDROs in transfer patients [8, 20]. The fact that most screenings yielded resistant Gram-negative bacteria is also consistent with recently published data [8, 11]. The very low rate of MRSA detections, however, is unexpected given the higher rates of MRSA abroad, as suggested by data from the European Centre for Disease Prevention and Control [3].

A number of studies show that traveling to certain foreign countries is a risk factor for acquiring MDRO colonization [24–27]. In these studies travel to India and the African continent stood out for the elevated rates of such colonization. India, in particular, is notable for being the country where the NDM-1 resistance gene first surfaced [13]. Many other Asian countries are affected by this development. Our data confirm that patients returning from Asia were more likely to be colonized than those returning from other regions. In contrast to a study from the Netherlands, southern Europe could not be linked to MDRO colonization in our work, even though there is a gradient of Gram-negative resistance from the Mediterranean area to Scandinavia [3, 11]. Transfer of a patient from outside Europe remained an independent factor for colonization in our analysis.

Our findings also show that transfer patients who received antibiotic treatment on admission were more likely to have a positive screening. This finding is not surprising. Even without an appropriate travel history, prior antibiotic use is a well-recognized risk factor for colonization and infection with MDROs [14, 28–31].

The Dutch study also meant to elucidate the association between patient-related factors and MDRO carriage in repatriated patients [11]. It is, however, an older study conducted between 1998 and 2001, when the global prevalence of MDROs, especially of Gram-negative bacteria, was much lower than today. This may be an explanation for why we identified additional risk factors. Skin lesions and a history of surgical procedure abroad, for example, were associated with having a positive screening in our study. To our knowledge this has not been recognized in returning patients before. However, our findings align with the commonality of risk factors for resistant pathogens first highlighted by Safdar and Maki in 2002 [31]. Patients who had an active infection on admission to our institution were also more likely to have a positive screening and this association remained in the multivariate model.

The standard screening set (nasal, inguinal swab, plus either a rectal swab or stool sample) was supplemented by additional swabs if certain clinical factors were present (skin lesions, urine in patients with urinary catheter, drainage fluid in a patient with wound drain in place, and tracheal secretions in intubated or tracheostomy patients). Based on this a transfer patient had at minimum of three screening samples taken. Those who turned out to be carriers had more screening samples taken (corresponding to the wounds or devices they had) than the non-carriers. In the literature, exposure to all types of invasive devices appears as a risk factor for MDRO acquisition [31].

The definition of transfer patients is not uniform in older studies. We defined transfer patients as patients who were exposed to a healthcare system abroad or in a high prevalence region in Switzerland over the past six months. In some studies the authors only captured patients who underwent direct transfer from a hospital abroad [8, 11], while others screened patients who were exposed over various time frames, e.g., the past 4 weeks [20] or 12 months [2, 15]. In our study, the longest interval between last healthcare exposure and presentation to our hospital was 126 days. We do not know if longer intervals were missed by the admitting services or if there were in fact none. The ideal time frame to incorporate distant healthcare exposure abroad remains unclear.

We screened all transfer patients on admission independent of whether they had been in- or outpatients abroad and found no association between screening positivity and the type of hospital exposure. We could not identify other studies that made this comparison. Kaiser and colleagues [11] found no association between duration of stay at a hospital abroad and MDRO colonization, which supports our findings. Thus, it appears that the length of hospitalization abroad has limited influence on the colonization status, just like type of exposure has. While some studies show that contact to a healthcare institution or a transfer from abroad is a risk factor [14, 20], others demonstrate that healthy travelers who were never hospitalized abroad were found to carry MDROs when they returned home [24–27]. It is unclear if a stay in a hospital abroad is a more relevant determinant of MDRO acquisition than a visit in a high endemic country. More studies are necessary to answer this question.

The original aim of the study was to elicit factors that could help us improve our screening strategy. Had we limited the screening to those with risk factors (hospitalization outside Europe, history of surgical procedure in the hospital abroad and – on admission to our hospital - active infection, presence of skin lesion and antibiotic treatment), we would have swabbed only 61.7 % (145 patients) of all transfer patients. With this “risk factor based screening” we would have found 36 patients with multiresistant Gram-negative bacteria, representing 86 % of all carriers we identified with the current strategy.

A standard screening set costs approximately 118 Swiss Francs. For 235 screenings, assuming standard triple sets, we spent 27`730 Swiss Francs. This is 660 Swiss Francs per identified carrier. For comparison, the cost of the alternative strategy would have been 475 Swiss Francs. Modifying our strategy to only screen patients with ≥ 1 out of five risk factors would have meant missing six colonized patients. A detailed cost-benefit analysis, however, would need to take secondary costs such as clinical infection and transmission to others into account.

Our study has some limitations. We did not conduct admission screening for all patients and cannot make a statement on baseline MDRO colonization in our patient population. Moreover, we cannot be sure that every patient who had a contact to a healthcare system abroad was screened on admission. This depended on the admitting service and their thoroughness when taking the history. Because of the missing or insufficient documentation of patient histories from the hospitals abroad we were only able to analyze antibiotic treatment on admission in our hospital and could not collect sufficient data on previous antibiotic treatment. Also, it was not possible for us to determine how long their stay in the hospital abroad was if patients had been inpatients there. As we screened patients on admission to our hospital we also cannot be sure if patients acquired the MDRO in the hospital abroad or if they were colonized with these bacteria before traveling to the foreign country.

## Conclusion

Guidelines recommend to screen patients returning after exposure to a healthcare system abroad. We screened patients who were exposed to healthcare abroad or in a high prevalence region in Switzerland over the past six months assuming they were at risk for being colonized with MDROs. Eighteen percent of these patients were screened positive on admission. The major risk factors for acquiring a Gram-negative MDRO was hospitalization outside of Europe (*p* < 0.001), history of surgical procedure in the hospital abroad (*p* = 0.007), and on admission to our hospital active infection (*p* = 0.002), antibiotic treatment (*p* = 0.014) and presence of skin lesions (*p* = 0.001). Only hospitalization outside of Europe and active infection on admission remained as independent predictors.

Our data suggest that a large proportion of patients (i.e., 82 %) transferred to Switzerland from hospitals in high MDRO prevalence areas are unnecessarily screened for MDRO colonization. Basing our screening strategy on certain criteria (such as presence of skin lesion, active infection, antibiotic treatment, history of surgical procedure abroad and hospitalization outside of Europe) could be a more cost-effective strategy.

## Key points

We performed a screening study of and determined risk factors for multi-drug resistant organism (MDRO) colonization in patients transferred from abroad. Independent risk factors for being MDRO colonized were hospitalization outside of Europe, surgical procedure in the hospital abroad, active infection at time of admission, antibiotic treatment on admission and skin lesions on admission.
